# Occurrence and Diversity of CTX-M-Producing *Escherichia coli* From the Seine River

**DOI:** 10.3389/fmicb.2020.603578

**Published:** 2020-12-09

**Authors:** Delphine Girlich, Rémy A. Bonnin, Thierry Naas

**Affiliations:** Team Resist, UMR1184, LabEx Lermit, Bacteriology-Hygiene unit, APHP, Hôpital Bicêtre, Université Paris-Saclay, Le Kremlin-Bicêtre, France

**Keywords:** CTX-M-14, CTX-M-15, *Escherichia coli*, Seine river, plasmids

## Abstract

CTX-M-producing *Escherichia coli* are spreading since 1999 both in clinical and in community settings. Environmental samples such as rivers have also been pointed out as being vectors for ESBL producers. In this report, we have investigated the presence and the diversity of CTX-M-producing *E. coli* isolates in two samplings of the Seine River (next to Notre Dame), Paris France, performed in June 2016 and 2017. The total number of bacteria growing on the selective ChromID ESBL agar was 3.1 × 10^5^ cfu/L (23.8% of all growing bacteria) in 2016, whereas it was 100-fold lower in 2017 (3 × 10^3^ cfu/L; 8.3% of all growing bacteria). However, among them, the prevalence of ESBL-producing *E. coli* increased from <0.1 to 1.1% in one-year. ESBLs were exclusively of the CTX-M-type: CTX-M-1 (*n* = 5), CTX-M-15 (*n* = 7), CTX-M-14 (*n* = 1), and CTX-M-27 (*n* = 2). The isolates belonged to several multi locus sequence types, and a wide diversity of incompatibility groups of plasmids were identified in those *E. coli* isolates. The occurrence and diversity of *E. coli* isolates belonging to many clones and producing many CTX-M-variants have been identified in our study. The presence of these bacteria in rivers that are open again for recreational usage (swimming) is worrying as it may contribute to further dissemination of ESBL producers in the community.

## Introduction

*Escherichia coli* is an ubiquitous human pathogen, most commonly involved in urinary tract infections and bacteremia in humans and animals (Rogers et al., 2011). Plasmid-mediated extended-spectrum β-lactamases (ESBLs) have become predominant in community-onset *E. coli* infection (Pitout et al., 2005). The first human CTX-M variant (previously named MEN-1) was initially reported in 1991 from a clinical *E. coli* isolate from France (Bernard et al., 1992). Since, CTX-M-producing *E. coli* have increasingly spread both in hospitals and in the community (Cantón et al., 2012) and represent now the most prevalent ESBLs worldwide. They are divided into five groups based on amino acid sequence: the CTX-M-1, CTX-M-2, CTX-M-8, CTX-M-9, and CTX-M-25 groups (Cantón et al., 2012).

The CTX-M-15 variant from the CTX-M-1 group, was first described in 2001 from several enterobacterial isolates from India on large-sized plasmids along with an IS*Ecp1* insertion sequence upstream of the *bla*_*CTX–M–*__15_ gene (Karim et al., 2001). Since 2008 (Coque et al., 2008), it has rapidly become the most prevalent ESBL worldwide in humans, especially linked to an *E. coli* group B2, serogroup O25b, sequence type 131 (ST131). A collection from eight European countries demonstrated also the presence of ST131, including 6% of ESBL-producing *E. coli* isolates recovered from companion animals (Ewers et al., 2010). The water environment is conducive to the transfer of resistance genes between species, where ESBL producers from various sources get in contact with a broad range of potential recipients. A previous study, conducted in 2011, reported occurrence and diversity of ESBL-producing *Aeromonas* spp. in the Seine river (Girlich et al., 2011). However, at this date, no ESBL-producing *E. coli* isolates were identified in those samples, and the presence of CTX-M *E. coli* producers in rivers was still an exceptional event as shown by Kim et al. (2008) in Korea in 2008 or by Dhanji et al. (2011) in the United Kingdom in 2011. More recently, the emergence of ESBL-producing *E. coli* occurred in urban rivers. In Austria, CTX-M-producing *E. coli* were identified in the River Mur in the center of Graz, Austria’s second largest city (Zarfel et al., 2017). In Guadeloupe, the predominance of CTX-M-producing *E. coli* has been reported from waste water treatment plant effluents (Guyomard-Rabenirina et al., 2017). In contrast to what was reported from European countries, the occurrence of CTX-M-*E. coli* producers was high in the Pearl River in China (Ye et al., 2017).

The aim of the present study was to investigate the presence of expanded-spectrum cephalosporin (ESC)-resistant *E. coli* isolates in the water of the Seine River, Paris, France, sampled in June of two consecutive years (2016 and 2017) at the same centrally located sampling spot (next to the Notre Dame). We report here, the isolation of CTX-M-type ESBL-producing *E. coli* isolates and the in-depth genomic characterization of 15 of them.

## Materials and Methods

### Water Sampling, ESBL Detection, and Plasmids

Sampling of the Seine River water, Paris, France, was performed in June of two consecutive years (2016 and 2017) at the same centrally located sampling spot (next to the Notre Dame). Samples were collected *c.a.* 1 m from the shore and *c.a.* 20 cm below the water surface using a 1-L sterile plastic bottle connected to a rope. The bottle was immediately closed, transferred on ice to the bacteriology laboratory of the Bicêtre Hospital, Le Kremlin-Bicêtre, France, and directly processed upon arrival. Four hundred milliliters of water was filtrated through a nitrocellulose membrane (0.45 μm, Millipore), and the bacteria were resuspended from the membranes in 2 ml of sterile water. Aliquots (100 μl) were then plated on ChromID ESBL plates (bioMérieux, Marcy l’Etoile, France). Pink-colored colonies growing ChromID ESBL were identified by mass spectrometry (MALDI-TOF, Bruker, France), and the ESBL phenotype was evidenced by a double disk synergy test (Karim et al., 2001). Plasmids, extracted by the Kieser method were electroporated into *E. coli* Top10, as previously described (Girlich et al., 2011). In case electroporation did not work, mating out assay was performed as previously described (Girlich et al., 2011). Transformants or transconjugants were selected on cefotaxime (0.5 μg/ml) agar. Identification of replicon types of the plasmid incompatibility (Inc) groups was performed by PCR as previously described by Carattoli et al. (Carattoli, 2009). Using this typing scheme, 18 Inc groups may be identified: Hl1, Hl2, I1-Iγ, X, L/M, N, FIA, FIB, W, Y, P, FIC, A/C, T, FIIAs, F, K, and B/O.

### Rapid Identification of ESBLs

NG-Test CTXM-Multi, a rapid Lateral Flow Immuno Assay (LFIA, NG-Biotech, Guipry, France) was used to detect all five CTX-M-groups, as previously described (Bernabeu et al., 2020). Briefly, one colony was resuspended in the extraction buffer, vortexed, and 100 μl was dropped on the LFIA strip. Results were eye read after 15 min of migration.

### Genetic Analyses

Whole genome sequencing was performed on 15 selected ESBL-producing *E. coli* isolates using Illumina technology on a Nextseq 500 sequencer as previously described (Dabos et al., 2019). *De novo* assembly was performed by CLC Genomics Workbench v7.0.4 (Qiagen, Les Ulis, France) after quality trimming (Qs ≥ 20). The acquired antimicrobial resistance genes were identified using ResFinder (Bortolaia et al., 2020), incompatibility groups of plasmids were determined using Plasmid finder (Clausen et al., 2018), and the sequence type was obtained using the Multi Locus Sequence Typing (MLST) modules of the Center for Genomic Epidemiology with genes *adk*, *fumC*, *gyrB*, *icd*, *mdh*, *purA*, and *recA*^1^ (Larsen et al., 2012).

## Results

### Bacterial Counts and ESBLs

Total bacterial count on Mueller Hinton agar was 1.3 × 10^6^ cfu/L of Seine water samples in 2016, whereas it was 3.5 × 10^4^ cfu/L in 2017. Bacterial count growing on ChromID ESBL agar was 3.1 × 10^5^ cfu/L (23.8% of all growing bacteria) in 2016, whereas it was 100-fold lower in 2017 (3 × 10^3^ cfu/L; 8.3% of all growing bacteria). ESBL-producing *E. coli* isolates recovered were 11 per 100 ml and 4 per 500 ml of water, in 2016 and 2017, respectively. Among the total bacteria growing on ChromID ESBL agar, <0.1% (2.7 × 10^2^ cfu/L) were ESBL-producing *E. coli* in the samples from June 2016, whereas 1.1% (33 cfu/L) ESBL-producing *E. coli* isolates were identified in May 2017. ESBLs produced by the *E. coli* isolates were exclusively CTX-M enzymes (Table 1). NG-Test CTX-M-gr1, a LFIA specific for group 1 CTX-M-β-lactamases gave positive results for 12/15 ESBL*-*producing *E. coli* isolates.

**TABLE 1:** Genetic characteristic of ESBL-producing *E. coli* isolates from the Seine river from 2016 and 2017.

Isolate	Acquired resistance determinants	Serogroup	ST	Inc groups	Inc group of pCTX-M^a^
S46^*b*^	***bla*_*CTX–M–*__15_**, ***bla*_*OXA–*__1_**, ***bla*_*DHA–*__1_**, *aac(6*′*)Ib-cr*, *qnrB4*, *aadA5*, *catB3*, *mph(A)*, *sul1*, *tetB*, and *dfrA17*.	O101, H10-like	**617**	IncFIA + IncFIB + IncFII + Col 156 + ColMG + ColpVC	IncFIA + IncFIB
S47	***bla*_*CTX–M–*__15_**, ***bla*_*OXA–*__1_**, *strA*, *aac(6*′*)Ib-cr*, *aac*(3)*-Iia*, *strB*, *aadA5*, *catB3*, *mph(A)*, *sul1*, *sul2*, *tetA*, and *dfrA17*.	O102-like, H6-like	**405**	IncFIA + IncFIB + IncFII + Col BS	IncFIA + IncFIB
S55	***bla*_*CTX–M–*__15_**, *aadA5*, *mphA*, *sul1*, *tetA*, and *dfrA17*.	O?^*c*^, H9-like	**410**	IncFIA + IncFIB + IncFII + Col 156	IncFIA + IncFIB
S56	***bla*_*CTX–M–*__1_**, ***bla*_*TEM–*__52_,** *aadA17*, *aadA5*, *qnrS1*, *lnu(F)*, *sul2*, *dfrA1*, and *dfrA14*.	O8-like, H19-like	**162**	IncFIA + IncFIB + IncI1 + IncN + IncX1 + p0111	IncI1
S57	***bla*_*CTX–M–*__1_**	O?, H1	**104**	IncI1 + IncX4 + Inc X1	IncI1
S58	***bla*_*CTX–M–*__14_**	O25-like, H4	**131**	IncI1 + Col156 + IncFIA + IncFIB	ND^*d*^
S59	***bla*_*CTX–M–*__1_**, *strB*, *aph(3*′*)-Ia*, *strA*, *sul2*, and *tetA*.	O80, H45-like	**4175**	IncFII + IncY + IncFIA + IncFIB + IncQ	ND
S61	***bla*_*CTX–M–*__1_**, *aadA17*, *lnu(F)*, *sul1*, *sul2*, *tetA*, and *dfrA1*.	O9-like, H19-like	**162**	IncFIB + IncFIC	IncFIC
S65	***bla*_*CTX–M–*__27_**	O25-like, H4	**131**	IncI1 + Col156 + IncFIA + IncFIB + IncFII	IncFIA + IncFIB
S66	***bla*_*CTX–M–*__15_**, ***bla*_*TEM–*__1_,** *aac*(3)*-IId*, *aadA5*, *qnrS1*, *mphA*, *sul1*, *tetA*, and *dfrA17*.	O?, H8-like	**13**	IncB/OKZ + Col156 + IncFII	IncOKZ
S67	***bla*_*CTX–M–*__15_**	O25-like, H4	**131**	IncFIA + IncFIB + IncFII	IncFIA + IncFIB
S17-1	***bla*_*CTX–M–*__15_**, ***bla*_*TEM–*__1_,** *strA*, *aac*(3)*-IId*, *aadA5*, *strB*, *mphA*, *sul1*, *sul2*, *tetA*, and *dfrA17*.	O16, H5-like	**131**	IncFII + Col156 + IncFIB	ND
S17-2	***bla*_*CTX–M–*__27_**	025-like, H4	**131**	IncFIA + IncFIB + IncFII + Col 156 + ColMG + ColpVC	IncFIA + IncFIB
S17-3	***bla*_*CTX–M–*__1_**, ***bla*_*TEM–*__1_,** *aadB*, *aadA5*, *aadA1*, *floR*, *sul1*, *sul2*, and *dfrA17*.	O9-like, H25	**58**	IncI1 + IncFIB + IncFIC + IncFII	ND
S17-4	***bla*_*CTX–M–*__15_**, *aadA5*, *qnrS1*, *mphA*, *sul1*, and *dfrA17*.	O6, H16-like	**4**	IncFII + IncFIB	ND

*^*a*^Acquired resistance determinants, in bold are β-lactamase genes. ^*b*^Samples numbered as “S#” are samples collected in 2016; samples numbered as “S17-#” are samples collected in 2017. ^*c*^O?, is a non-typable O serogroup by using NGS tools. ^*d*^ND, not determined. PCR amplification with previously reported primers of the most currently described Inc families remained negative (Carattoli, 2009).*

### Resistome Analyses

WGS identified *bla*_*CTX–M–*__1_ (*n* = 5), *bla*_*CTX–M–*__15_ (*n* = 7), *bla*_*CTX–M–*__14_ (*n* = 1), *bla*_*CTX–M–*__27_ (*n* = 2), *bla*_*TEM–*__52_ (*n* = 1) ESBL genes, *bla*_*DHA–*__1_ (*n* = 1) cephalosporinase gene, and *bla*_*TEM–*__1_ (*n* = 3) and *bla*_*OXA–*__1_ (*n* = 2) penicillinase genes (Table 1). The results of the WGS were in accordance with those of the NG-Test CTX-M- MULTI LFIA, validating this latest test for the rapid detection of the five groups of CTX-M-producing Enterobacterales, as previously reported (Bernabeu et al., 2020). As observed in other studies, group 1 CTX-M-producing *E. coli* isolates were dominant in our study (Zarfel et al., 2017; Hooban et al., 2020).

As commonly found in CTX-M-producers, most isolates were multidrug resistant, possessing aminoglycoside-modifying enzyme [e.g., *aac(6*′*)-Ib*, *aadA1*, *aadA5*, or *aph(3*′*)-Ia*], quinolone-resistance genes (*qnrS1*), tetracycline (*tetA*, *tetB*), chloramphenicol (*catB3*), and trimethoprim/sulfamethoxazole (*dfrA14*, *dfrA17*, *sul1*, and *sul2*). Those resistance gene are, for most of them, carried by a class 1 integron (Table 1; Cantón et al., 2012).

### Clonal Relationship

**FIGURE 1:**
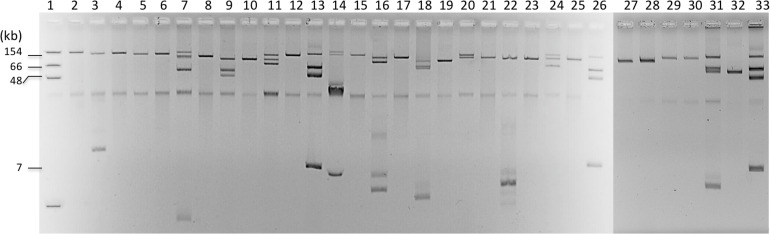
Phylogenetic tree of extended-spectrum β-lactamase (ESBL)-producing *Escherichia coli* from Seine River. The phylogeny was performed using CSIPhylogeny (https://cge.cbs.dtu.dk/services/CSIPhylogeny/). Year of isolation is indicated by colored circles and broad-spectrum β-lactamases by colored pentagons.

Clonal relationship of these isolates was initially assessed by MLST and then by WGS SNP analysis. MLST analysis revealed a wide diversity of clonal groups with 10 different STs among the 15 isolates. Noticeably, only two STs were represented with at least two isolates being ST162 (*n* = 2) and ST131 (*n* = 5) (Figure 1). WGS-based phylogeny confirmed this diversity but also indicated that the five ST131 isolates can be divided into two subclones (Figure 1). It can be noticed that ESBL distribution did not follow the clonal relationship. For instance, in ST131 isolates, three types of ESBLs were identified: *bla*_*CTX–M–*__14_, *bla*_*CTX–M–*__15_, and *bla*_*CTX–M–*__27_ genes. The wide diversity of clones may reflect the large spread of ESBLs in the community. Indeed, we did not identify a clonal spread of ESBL-producing *E. coli* but rather unrelated isolates that are present in the Seine River. Among the five ST131 *E. coli* isolates, four were genetically close (Figure 1). However, these isolates did not share the same resistome indicating that ST131 is widely distributed independent of the ESBL content as previously observed (Pitout and Finn, 2020). Moreover, the two closest ST131 (S65 and S17-2) possessing the *bla*_*CTX–M–*__27_ ESBL gene were recovered one-year apart indicating the persistence or continuing contamination by this clone.

### Plasmid Analysis

**FIGURE 2:**
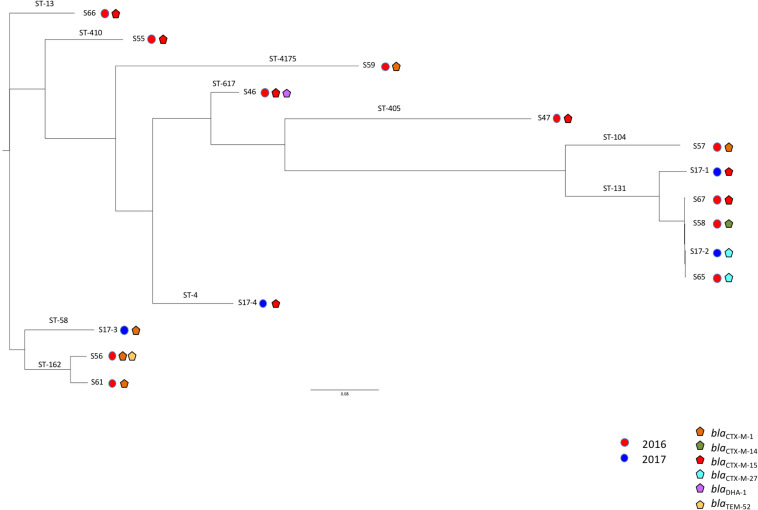
Plasmid extractions from cultures of the different isolates and their transconjugants or transformants. Lanes 1, 3, 5, 7, 9, 11, 14, 16, 18, 20, 22, and 24 correspond to S46, S47, S55, S56, S57, S59, S61, S65, S66, 17.1, S17.2, S17.3, S58, S59, and S17.4; Lanes 2, 4, 6, 8, 10, 12, 15, 17, 19, 21, 23, 25, 28, 30, and 32 correspond to their respective *E. coli* transformants or transconjugants; Lanes 13, 26, and 33 correspond to *E. coli* 50192 harboring four plasmids: 7, 48, 66, and 154 kb.

The *bla*_*CTX–M*_ genes were located on large plasmids of different sizes (Figure 2) belonging to diverse incompatibility groups (Table 1). Several studies have shown that plasmids of the IncF family were the predominant group that carry the *bla*_*CTX–M–*__15_ gene, whereas the *bla*_*CTX–M–*__14_ gene is carried on a variety of plasmid types, including on IncF, especially in the Far-East, and on IncK, in Western Europe (Bevan et al., 2017). Horizontal transfer of antimicrobial resistance plasmids by conjugation in Enterobacterales occurs in the human gut, animals, and the environment (Bevan et al., 2017; Zarfel et al., 2017). As previously reported, the main ESBL types identified in companion animals were CTX-M-14 (26.8%), CTX-M-15 (24.4%), CTX-M-27 (19.5%), and CTX-M-55 (19.5%) (Kawamura et al., 2017), and the most prevalent STs were ST131 (*n* = 15, 35.7%), followed by ST38, ST10, and ST410 (Kawamura et al., 2017). For example, among those STs, ST10/CC10 corresponds to an international cluster already identified in humans, wildlife infections, domestic farm animals, companion animals, and commercial chicken meat (Nascimento et al., 2017).

## Discussion

We identified in this study the occurrence of different ESBL-producing *E. coli* isolates from the Seine River in Paris, France. A wide diversity of clones was identified here. The most prevalent, with 4 isolates out of 15, was ST131. This result is not surprising given its widespread occurrence, but the presence of different CTX-M-variants belonging to different groups of enzymes was unexpected, as ST131 is frequently associated to CTX-M-15. We also identified one ST410 isolate. This clone has recently attracted not only the light by its association with the spread of the carbapenemase OXA-181 (Patiño-Navarrete et al., 2020) but also for its isolation in animals (Yang et al., 2019). Of note, two isolates of ST162 were recovered in this study. This clone has been reported to be associated with the resistance gene in wild avian isolates (Oteo et al., 2018). However, it remains difficult to conclude on the original source of these isolates, which could be of avian/environmental or human sources. Hooban et al. (2020) reported that most of the ESBL producers identified in aquatic environments around the world between 2010 and 2017 expressed *bla*_*CTX–M*_ genes (*n* = 21 among 29 studies), followed by *bla*_*TEM*_ (*n* = 18), and *bla*_*SHV*_ (*n* = 11). Surprisingly, among eight Chinese studies, only three identified CTX-M-producing *E. coli* isolates in rivers and lakes (Hooban et al., 2020). The prevalence of ESBL producers among waterborne thermo-tolerant coliforms ranged in amount from 11% (Ye et al., 2017) to 17% in Chinese rivers (Chen et al., 2010). Ye et al. (2017) identified only CTX-M-variants as ESBLs, with additional variants: i.e., CTX-M-55 and CTX-M-65 in addition to CTX-M-14 and CTX-M-15. Notably, a previous study, 6 years earlier, reported TEM (37.6%) and SHV (84.1%) as being the most common ESBL among clinical isolates from the same city of Chongqing in 2004 (Chen et al., 2010). In Brazil, four studies reported the presence of CTX-M but also of KPC-2 carbapenemase *K. pneumoniae* producers in rivers, lakes, and sea water (Hooban et al., 2020). The presence of *bla*_*CTX–M*_ genes in water is more and more frequent worldwide, most often associated with highly self-transferable plasmids. In all cases, it is likely that the transfer of these bacteria from the sewage to the rivers occurred. Most worrying, concomitant spread of carbapenemase genes has been witnessed in many countries including Switzerland, Spain, Portugal, Austria, United States, Brazil, India, and China (Hooban et al., 2020).

## Conclusion

The epidemic dissemination of CTX-M-encoding genes is largely due to their localizations on mobile genetic elements, such as plasmids, transposons, and integrons, which allow these genes to easily spread among bacterial communities (Cantón et al., 2012). In 2016, the samples were collected a few days after floods that occurred between the end of May and the beginning of June 2016, thus explaining a high prevalence of ESBL-*E. coli* isolates in the Seine river that likely originated from animal feces that have been drained by the rains. However, the presence and diversity of those isolates one-year later is more worrying, as it indicates a persistent contamination of the Seine river with ESBLs-producing *E. coli* isolates. This is especially worrying given that the many rivers all over Europe open again for different recreational and sporting activities, including swimming.

## Data Availability Statement

The datasets generated for this study can be found in NCBI BioProject, NCBI Accession No. PRJNA662045.

## Author Contributions

TN: conception, data analysis, writing, and proof-reading. RB: data analysis and proof-reading. DG: experimental work, data analysis, writing, and proof-reading. All authors contributed to the article and approved the submitted version.

## Conflict of Interest

The authors declare that the research was conducted in the absence of any commercial or financial relationships that could be construed as a potential conflict of interest.

## Footnotes

1 http://genomicepidemiology.org/
